# Biomolecular condensates as cellular memory modules: Thermodynamic principles and plant stress adaptation

**DOI:** 10.1016/j.bpj.2025.11.2681

**Published:** 2025-11-26

**Authors:** Sukhendu Maity, Panagiotis Nikolaou Moschou

**Affiliations:** 1 Department of Biology, University of Crete, Heraklion, Greece; 2 Institute of Molecular Biology and Biotechnology, Foundation for Research and Technology-Hellas, Heraklion, Greece; 3 Department of Molecular Sciences, Uppsala BioCenter, Swedish University of Agricultural Sciences and Linnean Center for Plant Biology, Uppsala, Sweden

## Abstract

Organisms frequently encounter abiotic stresses such as drought, salinity, and extreme temperatures, requiring sophisticated adaptive mechanisms. Stress memory enables them to respond more efficiently to repeated environmental challenges by retaining information from prior exposures. Biomolecular condensates, dynamic, membraneless cellular assemblies formed by liquid-liquid phase separation, have emerged as crucial regulators of post-transcriptional gene expression, particularly in stress conditions. These condensates modulate RNA fate and translational repression by selectively storing and organizing key molecules in ways that may contribute to cellular memory mechanisms. Here, we explore the biophysical principles underpinning condensate formation and dynamics, with a focus on processing bodies (PBs) as potential cellular memory storage systems. We propose a framework for how PBs might integrate biochemical and biophysical signals to encode, maintain, and retrieve stress-responsive information, and discuss the evidence supporting their role in coordinated stress responses and adaptive resilience in plants.

## Significance

Noninherited cellular memory, the ability to remember and respond more effectively to recurring stress, is critical for survival, yet how cells physically encode, retrieve, and erase this information remains unclear. This review proposes that biomolecular condensates function as dynamic memory storage systems. By integrating thermodynamic principles with kinetic modeling, we demonstrate how the condensates known as “processing bodies” encode stress history through molecular sequestration, maintain information via gel-like networks, and erase memory through regulated dissolution. We introduce a quantitative framework that transforms condensates from passive assemblies into optimized nonequilibrium information processors. This work reveals a previously underappreciated physical mechanism of cellular adaptation and provides testable predictions for understanding how organisms achieve stress resilience.

## Introduction

Cellular memory is broadly classified into somatic memory, which is maintained within a single organism’s lifespan, and transgenerational memory, which is transmitted to subsequent generations. Cellular memory enhances the response to recurring stress by retaining molecular traces of prior exposure (1). A prominent form of cellular memory is priming, whereby a mild or sublethal exposure to abiotic or biotic stress prepares the organism for a faster and more robust response upon reexposure to the same stressor (2). Priming induces diverse molecular changes including genetic modifications, transcriptional memory, stabilized proteins, and altered metabolic states that enable more efficient activation of stress-responsive genes and pathways during subsequent encounters (3). Among these, epigenetic modifications, heritable changes on DNA that do not alter the nucleotide sequence, such as DNA methylation, histone modifications, and chromatin remodeling, can persist through development or even across generations, underpinning transgenerational stress memory (4).

Analogous to membrane-bound compartments, liquid-liquid phase separation (LLPS) within living cells provides a physical mechanism to concentrate specific biomolecules in biomolecular condensates or “condensates,” excluding others, thereby orchestrating order amid cellular complexity. These membraneless assemblies arise primarily through multivalent interactions among proteins and nucleic acids, which drive molecules to demix into dynamic phases that differ from their surroundings in viscosity, density, and molecular concentration (5). Hence, condensates have the ability to “engulf” and retain information, which can be used as cellular memory or a priming mechanism for stress. Condensate formation is typically driven by scaffold proteins enriched in intrinsically disordered regions (IDRs) and modular domains, alongside nucleic acids (6,7,8). Initially, condensates often display liquid-like properties, resembling droplets; however, over time, their material properties can transition toward more solid or gel-like states, losing droplet-like behavior. These material transitions modulate internal dynamics such as molecular mobility and exchange rates, thereby influencing residence times of proteins and RNAs within condensates (9,10,11,12,13,14). Condensates can thus serve as organized storage depots for proteins and RNAs, with solid-like states reducing molecular accessibility and processing but increasing stability and storability (10). In plants, condensates regulate diverse developmental processes and stress responses (15,16). Condensates may be constitutive or induced by specific environmental cues and include assemblies such as stress granules (SGs) (17), processing bodies (PBs) (18), nucleoli (19), and Cajal bodies (20,21). The critical role of condensates in plant stress responses has been increasingly recognized, with recent comprehensive reviews highlighting how condensates enable plants to sense, transduce, and adapt to environmental challenges (22).

In this review, we focus on cytoplasmic condensates as mediators of cellular memory for rapid and adaptive stress responses, using PBs as a representative example. PBs are omnipresent, allowing their use as memory storage hubs; they are also easily tracked and aggregate proteins and RNAs (23,24,25). Key PB components include RNA decay enzymes such as the decapping proteins decapping protein 1 (DCP1) and DCP2, with accessory factors such as DCP5 and VARICOSE (VCS), exonucleases including 5′-3′ exoribonuclease 4 (XRN4), RNA helicases, and diverse canonical and noncanonical RNA-binding proteins (RBPs) involved in mRNA storage, silencing, and degradation (26,27,28,29,30,31). Some noncanonical RBPs, often termed moonlighting enzymes (32) primarily function in metabolism, signaling, or structure but also bind specific RNA sequences within PBs to regulate certain transcripts. PBs function as hubs for mRNA triage, where transcripts can be temporarily stored in a translationally repressed state or targeted for decay, typically initiated by decapping (5′ removal of RNA cap) or removal of poly(A) tails (known also as deadenylation) (10,33,34,35). Furthermore, PBs participate in microRNA-mediated silencing pathways and dynamically regulate mRNA fate during stress, making them essential regulators of RNA metabolism and cellular homeostasis (30,36,37,38). We discuss below how such features of PBs, and condensates broadly, bestow them with remarkable capabilities as storage depots for cellular memory.

## How are the thermodynamics of condensates linked to memory?

Condensates exhibit dynamic behaviors such as flowing, fusion, fission, and component exchange with their surroundings (e.g., nucleoplasm or cytoplasm). These dynamics are vital for maintaining cellular memory by enabling the storage, accumulation, and controlled release of molecular information. From a thermodynamic perspective, condensate formation is a nonequilibrium process (39,40), a constant state of flux that keeps the system away from thermodynamic rest. The process also includes nucleation (scaffolding), growth, and coarsening (41), wherein smaller droplets shrink as larger ones grow over time. This characterization may initially seem counterintuitive when compared with simplified equilibrium-based models of LLPS. Hence, processes such as nucleation can result from spontaneous demixing in systems at or near equilibrium, and coarsening typically reduces interfacial energy by driving the system toward equilibrium. However, in living cells, condensate dynamics are fundamentally nonequilibrium processes driven by active cellular metabolism and regulation. Unlike idealized physicochemical systems, biological condensates are continuously subjected to ATP-dependent remodeling, enzymatic modifications, active transport, and metabolic regulation that constantly perturb the system. These active processes, including post-transcriptional and post-translational modifications (PTMs), concentration changes driven by transcription and degradation, and environmental fluctuations, ensure that condensates never settle into true thermodynamic equilibrium (i.e., steady state). Instead, they occupy dynamic steady states characterized by continuous assembly, disassembly, and compositional remodeling. This nonequilibrium nature is not merely a complication but a feature: it endows condensates with the responsiveness and adaptability necessary to function as cellular memory modules, capable of encoding, storing, and erasing information in response to changing environmental conditions. These dynamics can be modulated by environmental changes that can affect PTMs, or alteration of concentrations of molecules within or outside of condensates (42,43,44,45,46).

**Figure 1:**
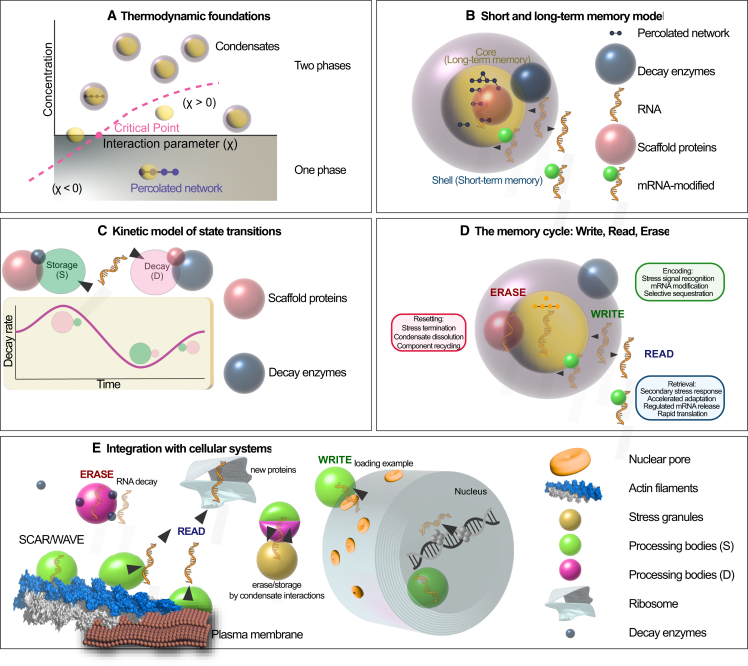
Biophysical framework for condensate-mediated RNA memory. (*A*) Thermodynamic foundations of phase separation. The formation of biomolecular condensates is governed by the interplay of concentration and interaction strength. When the effective interaction parameter (χ) surpasses the critical threshold (e.g., a Csat), the system undergoes a phase transition from a homogeneous one-phase regime to a two-phase regime characterized by condensates coexisting with the dilute phase. Within this regime, percolated molecular networks stabilize the condensed phase (*yellowish cores*), which sometimes form cores with prepercolation networks, providing a physical incentive for selective compartmentalization of RNAs and proteins. The cores with precolated networks may precede the formation of condensates (*free yellowish cores*). (*B*) Core-shell organization as a short- and long-term memory module. Condensates can display mesoscale heterogeneity, often represented as core-shell structures. The shell behaves as a short-term memory compartment, facilitating rapid binding and release events (*black arrow direction*), while the core functions as a long-term memory reservoir, where reduced molecular mobility and stronger interactions extend the lifetime of stored RNAs and proteins. In the core, RNAs and other molecules can be entrapped from the shell, if they could form long-lived interactions with core proteins, such as RBPs and scaffold proteins. Scaffold proteins define network connectivity, RNA modifications alter binding free energies, and decay enzymes introduce active turnover, jointly tuning information persistence. Decay enzymes are most likely active in the shell region (or juxtaposed to the condensate), where mobilities are larger and relaxation dynamics fit enzymatic reactions (56,57). Importantly, the suggested material heterogeneity need not be strictly concentric; mixed or patchy distributions of components are frequently observed, reflecting local variations in interaction strengths and molecular crowding (58). (*C*) Kinetic model of state transitions. RNA fate reflects stochastic switching between storage (S) and decay (D) states, driven by dynamic interactions with scaffold proteins and decay enzymes. These processes are inherently out of equilibrium: scaffold-mediated stabilization decreases effective decay rates, while enzymatic activity accelerates degradation. The result is temporal modulation of decay kinetics, with oscillatory or nonmonotonic patterns reflecting the system’s ability to tune stability windows for information storage, which fits out-of-equilibrium patterns. (*D*) The memory cycle: write, read, erase. The information cycle emerges from physical transitions between metastable states. Write (*green*): condensates encode information by selectively sequestering mRNAs in response to stress or other stimuli. This involves stress signal recognition, mRNA and protein modification (e.g., m^6^A or restructuring), and selective recruitment into the condensate environment. These processes modulate accessibility and translational readiness, creating a molecular imprint of the stress episode. Read (*cyan*): following stress adaptation or upon secondary stress exposure, stored mRNAs can be rapidly retrieved from condensates. This enables accelerated reinitiation of translation, selective mRNA release, and swift mobilization of protein synthesis machinery, thereby ensuring efficient cellular adaptation. Condensates thus act as short-term memory hubs that enhance the kinetics and specificity of the response. Erase (*magenta*): condensates and their stored messages can be disassembled once signals abate. This involves condensate dissolution, component recycling, and RNA turnover, which reset the system and prevent inappropriate persistence of stress programs (note: not signaling). Erasure safeguards fidelity, ensuring that stress-induced memories do not interfere with future rounds of encoding and retrieval. Together, the write-read-erase cycle frames condensates as dynamic molecular archives that transiently encode, utilize, and reset stress-adaptive information. This cyclic framework highlights their role not as static storage bodies but as programmable regulators of RNA and protein (and likely metabolite) fate, coupling biophysical assembly principles with adaptive cellular physiology. (*E*) Integration with cellular systems. Condensate-based memory operates within a broader mesoscale network of cellular structures. SGs and PBs represent archetypal condensates that bias RNAs toward storage or decay paths (note the two-colored PBs), exhanging RNAs (likely at their surfaces). Ribosomes couple condensate release to translational output, while nuclear pores feed RNAs to PBs. Cytoskeletal scaffolds (e.g., SCAR-WAVE-driven actin filaments) link to the plasma membrane and can dissolve PBs through wetting processes on their surfaces and COAST actions, thereby releasing RNAs for active translation (*arrowheads* denote RNA tracks). Through this integration, write-read-erase cycles are embedded in a cellular context, ensuring that the biophysical rules of phase separation and kinetic control translate into adaptive responses to fluctuating environments. Overall, this framework positions RNA-protein condensates as nonequilibrium memory devices, where thermodynamic driving forces, kinetic regulation, and cellular architecture (i.e., cell shape and cytoplasmic dynamics) converge to encode, retain, and reset molecular information.

Initial views of condensates as ideal LLPS systems have been refined to incorporate observed heterogeneity, viscoelastic properties, and complex interaction networks within condensates. Modern frameworks capture how condensates encode both persistence and responsiveness, providing a more nuanced foundation for understanding stress memory. Key to this understanding is the Flory-Huggins model of polymer solution thermodynamics, where a critical concentration (Csat) defines the threshold at which phase separation occurs (8,21,47,48,49,50,51,52,53,54,55). Hence, Csat in practical terms defines the threshold at which a macromolecule-solvent mixture separates into two distinct phases: a macromolecule-rich phase (e.g., a condensate) and a macromolecule-poor phase (e.g., the surrounding cytoplasm), with the transition governed by the Flory-Huggins interaction parameter (χ) (Fig. 1 *A*). This parameter quantifies the energetic favorability of interactions between macromolecules and solvent, thus influencing whether phase separation occurs (59,60,61).

When χ > 0, macromolecule-solvent interactions are unfavorable. In this regime, macromolecules prefer to interact with each other rather than with the solvent, driving phase separation once their concentration exceeds Csat (62) (Fig. 1 *A*). A familiar example is oil in water: because oil-water interactions are unfavorable, oil molecules cluster into droplets instead of dispersing evenly. As a cautionary note, while simple examples such as oil-water separation illustrate the basic principle of unfavorable mixing, condensates, as mentioned above, often display various material properties that extend beyond classical LLPS (23). Yet, the implied “worse mixing” is directly analogous to macromolecules clumping together when χ > 0 (Fig. 1 *A*, two phases). Importantly, the Csat threshold acts as a molecular filter, reducing noise in information storage: only when concentration surpasses this critical point will condensates form or a molecule incorporated into condensates, ensuring that the cell selectively encodes molecules deemed significant enough to contribute to memory. By contrast, when χ < 0, macromolecule-solvent interactions are favorable, and molecules mix readily with the solvent (Fig. 1 *A*, one phase). A simple everyday example is sugar dissolving in water: sugar-water interactions are strong enough to keep the sugar evenly dispersed, preventing droplet formation. While, again, we would like to draw attention to the simplicity of the example used, biologically, this corresponds to conditions where proteins or RNAs remain soluble and diffuse, avoiding condensate formation. In this regime, memory is not encoded in condensates but remains in the soluble pool, allowing rapid turnover and flexibility.

At equilibrium, chemical potentials and osmotic pressures are equalized across coexisting phases, with the Csat defining the concentration of the dilute phase. In the framework of cell memory, this implies that PBs or other condensates persist only when the concentration of stress-responsive RNAs or RBPs surpasses this critical threshold. Above Csat, these molecules are selectively sequestered into condensates, which effectively “record” a cellular event by retaining key transcripts and proteins produced in this time snapshot. Conversely, when concentrations fall below Csat, condensate dissolution occurs, akin to an erasure event that resets memory. However, this reset is partial, as residual molecular signatures, such as chromatin modifications, RNA pools, or PTMs, can retain storability and prime the cell for more rapid or robust future responses.

Proteins with similar χ tend to have comparable Csat values, yet their functional roles and material properties (solid-like versus liquid-like states) may vary significantly (63). In terms of memory, while Csat controls the assembly of condensates, their material state critically shapes their stability and ability to retain molecular cargo over time. Therefore, the nuanced interplay between Csat and condensate material properties not only determines when condensates form, but could also govern the fidelity and durability of cellular memory (Fig. 1 *B*).

The identification of key polar residues, such as asparagine (Asn) and glutamine (Gln) within IDRs, as drivers of phase separation led to the development of the “stickers and spacers” model by Rosen and colleagues (5). These IDRs are commonly found in prion-like domains of RBPs such as the human model-condensating proteins heterogeneous nuclear ribonucleoprotein A (hnRNPA1) and fused in sarcoma (FUS) (64,65). This model integrates key principles, including LLPS, multivalency, and percolation. A percolation transition denotes a critical threshold at which local intermolecular connections form a spanning, system-wide network, establishing large-scale connectivity (66). Multivalent macromolecules undergo such transitions mediated by specific interactions among sticker motifs capable of hydrogen bonding, ionic interactions, π-π stacking, cation-π interactions, or hydrophobic contacts (67,68,69). The stickers and spacers framework conceptualizes stickers as residues mediating cohesive interactions interspersed with less interactive spacers, encoding both the phase behavior and material properties of condensates. Aromatic residues such as tyrosine (Tyr) and phenylalanine (Phe), along with polar residues Asn and Gln, are key stickers driving phase separation within IDRs. These residues often act synergistically with charged residues to modulate interaction strength and specificity.

Adding complexity, multivalent biomolecules can form small clusters or prepercolation oligomers even below the full-phase separation Csat concentration (23). These tiny, nascent structures, known as prepercolation clusters or higher-order oligomers, are not true condensates (Fig. 1 *A*, percolated networks in and out of the condensates). They lack a clear phase boundary or distinct internal environment, but they are crucial for what is to come. These nascent oligomeric structures represent early-stage assemblies that lack the characteristic phase boundary of mature condensates but serve as critical nucleation sites for subsequent condensate growth and maturation. Unlocking the secrets of these early assemblies is essential for understanding how the cell builds its complex, nonequilibrium compartments from the ground up and thus store or restore cellular memories.

Thermodynamic parameters, including molecular concentration and temperature, critically influence multivalent sticker interactions and thus regulate condensate size, number, and dynamics (15,23,70,71,72,73,74,75). Unlike stoichiometric and structurally rigid protein complexes such as ribosomes, condensates are nonstoichiometric assemblies whose formation and stability respond sensitively to these thermodynamic parameters. This responsiveness allows condensates to dynamically assemble or dissolve in reaction to changes in the cellular environment. A relatable daily example is how soap bubbles form and dissolve depending on factors such as soap concentration and temperature: when conditions are right, bubbles appear and persist; when conditions change, they burst and disappear. Similarly, within the context of memory, these principles suggest that the very same factors governing condensate assembly also control their ability to serve as dynamic storage compartments.

Typically, a small number of “scaffold molecules” (or nucleating molecules), typically multivalent proteins or RNA, initiate phase separation by forming a percolated network (73,76). “Client molecules,” which are usually less multivalent, are then recruited into this preformed network (5,13,72,77,78,79) (Fig. 1 *B*). Although not required for the onset of percolation, clients fine-tune the properties of the condensate by altering scaffold-scaffold connectivity. Depending on their valency and binding sites, clients may stabilize the network (by forming scaffold-client-scaffold bridges) or destabilize it (even dissolving it by competing for scaffold sites), thereby regulating condensate size, composition, and persistence (16). In this framework, scaffolds establish the baseline percolated network, usually in the center of the condensate (core), while clients modulate how robustly or flexibly that network stores information (in the “shell”; Fig. 1 *B*). Thus, scaffolds encode the structural backbone of cellular memory, while clients dynamically regulate memory strength, flexibility, or erasure.

Condensates can form in two fundamentally different ways. In the first way we discussed, molecules come together because they attract each other. But there is a second mechanism: molecules can separate into condensates simply because they cannot coexist; they actively avoid each other, like oil avoiding water. This “segregative” mechanism is called COAST (co-assembly through segregative transitions) (80). This process resembles but is not identical to what chemists call a eutectic transition, where a mixture crystallizes into two distinct solids upon cooling. However, here, we are borrowing the concept of “eutectic mixing ratios” to describe condensates: at certain optimal proportions, different molecular types can condense together more effectively, even without directly attracting each other (81,82). Why does this matter for memory? This segregative mechanism could allow a single condensate to contain multiple separated “memory compartments” (or even to have in single-cell compositionally distinct condensates), like having different colored sections in a notebook. Each compartment (or condensate) could store distinct types of information about different stresses or cellular states. During development or new stress encounters, the cell could selectively erase one compartment while preserving others, enabling sophisticated, multilayered memory storage and retrieval within the same condensate structure.

From the above, one can interpret LLPS as a primal density transition driven by solubility limits and unfavorable solvent interactions, producing phase coexistence but not necessarily a fully connected molecular network. In contrast, percolation reflects the formation of a continuous, gel-like network of molecular interactions, which can emerge transiently or stably even below Csat. In this way, percolation acts as an early organizing step that lowers the energetic barrier for condensate assembly and primes the system for LLPS (and thus writing a memory). Although the two are thermodynamically distinct, they are coupled processes, a relationship captured by the renormalized Flory-Huggins parameter (χ′ = χ + Δχ), where Δχ accounts for specific sticker-sticker interactions that enhance effective incompatibility with the solvent. The sequence of transitions is decisive: if the percolation threshold (Cperc) is crossed before saturation (Cperc < Csat), a macrogel spanning the system forms and suppresses droplet assembly, whereas if saturation occurs first (Csat < Cperc < Cdense), droplets nucleate by LLPS and only later undergo percolation inside the dense phase, producing viscoelastic microgels (23,55,59,73,75,83). Once LLPS initiates, however, the dense phase typically supports percolated networks that confer viscoelasticity, persistence, and nonideal liquid behavior. Because macromolecular conformations are environmentally sensitive and can display orientational ordering, condensates may exhibit gel-like, liquid-crystalline, or semicrystalline organizations across different length scales. Thus, percolation can be viewed as a rapid, microscopic organizer of molecular clusters, while LLPS represents the slower mesoscopic transition in which clusters coalesce into a distinct dense phase; yet, in living cells, where multivalent interactions and concentrations change dynamically, these processes often unfold nearly simultaneously, making condensates emergent materials whose properties arise from the continual interplay between percolation and phase separation.

The layered architecture of condensates (i.e., concentric core-shell, Fig. 1 *B*) can explain the behavior of PBs, nucleoli, and SGs, where liquid-like peripheries allow rapid molecular exchange, supporting “short-term memory,” while elastic or gel-like cores provide greater stability and “long-term memory.” Crucially, as mentioned above, there is a functional link between liquidity and storability: reduced liquidity correlates with increased molecular retention and storage capacity. LLPS primarily increases the number of clients (“memory capacity”) by concentrating molecules into the condensate phase through favorable macromolecule-solvent interactions. This promiscuity creates a shell compartment rich in diverse molecules that expands around the stable core. However, LLPS alone does not inherently require these client molecules to be physically interconnected, as they can simply be concentrated in the same phase without forming direct molecular linkages (84). Hence, the formation of percolated or gel-like networks within condensates enhances their ability to stably sequester specific transcripts and proteins, serving as durable repositories of molecular information. Therefore, percolation provides more interaction sites than LLPS alone by physically linking molecules into a network, but LLPS controls the overall recruitment and enrichment of molecules into condensates. The two processes often work together in biological condensates to regulate both client concentration (i.e., quality of information stored) and material state (i.e., quantity of information stored liquid-like versus gel-like). Thus, the interplay between phase separation and percolation (and likely COAST) creates a layered memory architecture, where shells act as dynamic buffers and cores as durable repositories. We propose that the shell regions, due to their larger geometric volume, can store numerous transient interactions (short-term memory), whereas the smaller, gel-like cores support more stable but less-complex long-term interactions (Fig. 1, *B* and *C*).

## From thermodynamic equilibrium to nonequilibrium dynamics: A framework for modeling memory

The thermodynamic principles of LLPS and percolation provide a static snapshot of the conditions necessary for condensate formation and stability. However, cellular memory is an inherently dynamic process. To bridge this gap and understand how PBs switch functional states in response to time-varying stress signals, we must move from equilibrium thermodynamics to nonequilibrium kinetic models. The assembly, disassembly, and compositional remodeling of PBs can be conceptualized as a stochastic process given the out-of-equilibrium context that is sensitive to the ongoing conditions (Fig. 1 *C*). We suggest that Markov property, where the future state depends only on the present state and not on the sequence of events that preceded it, is a reasonable first approximation for this system. This is justified because: 1) the molecular interactions within PBs (e.g., weak, multivalent bonds) have short relaxation times (nanoseconds) relative to the timescale of stress events (minutes to hours (73)) or 2) the primary “memory” of past stress is encoded in the current composition of the PB (e.g., the concentration of specific clients and scaffolds; as has been calculated by our group (10)), making the present state a sufficient descriptor for predicting immediate future behavior.

In this framework, a PB can occupy a set of discrete, coarse-grained functional states that can partially behave in a Markovian way (85). Transitions between these states occur probabilistically at rates modulated by environmental inputs such as stress intensity. This allows us to model how PBs probabilistically encode, maintain, and erase information. Following this rationale, we can model PB dynamics using a continuous-time Markov jump process; however, we recognize potential inaccuracies of the system as described below. For the sake of simplicity, let us define a simplified model with two core functional states: state S (storage), characterized by a high ratio of sequestered/stabilized transcripts to decay factors; state D (decay), characterized by the active engagement of the decay machinery (e.g., DCP1/2 interactions, XRN4 incorporation) and a higher rate of RNA degradation (Fig. 1 *C*). The time-dependent stress signal, I(t), modulates the transition rates between these states. The environment thus acts as an input variable, and the steady-state probability distribution over these states can be viewed as an output “classification,” decoding stimuli into functional decisions.

These nonequilibrium networks are subject to thermodynamic constraints limiting their ability to distinguish complex inputs or generate nonlinear responses. Architectures such as serial cascades arising from complex or prolonged stresses can produce switch-like transitions and richer functional outcomes in PBs and condensates. Importantly, LLPS systems are inherently out of equilibrium, and they benefit from input multiplicity to enhance responsiveness and functional expressivity (86). Stressors involve a reactive oxygen species burst, followed by additional contrasting inputs (antioxidants) during the stress course; this is expected to generate an oscillatory behavior of storage/decay with varying amplitude (Fig. 1 *C*). This perspective reinforces the notion that evolved phase-separating systems are not merely passive thermodynamic assemblies, but optimized information processors, shaped by the constraints and opportunities of nonequilibrium biophysics. Below, we provide a more mathematical incentive to this problem.

## Cell memory and possible mechanisms

Here, we focus on plants because, as they cannot flee changing environmental conditions and must instead “remember” past stresses to optimize their future responses. Daily cues, such as sudden temperature fluctuations from passing clouds, brief droughts, or transient light bursts, require plants to rapidly adjust their physiology or reject these cues altogether. This intrinsic need for adaptability makes plants an ideal system for studying how condensates encode, store, and interpret cellular memory. Critically, these processes can be framed within the thermodynamic principles detailed above, providing a robust conceptual basis to investigate cell memory.

Cellular memory mechanisms operate across multiple molecular layers, from chromatin modifications to post-transcriptional and post-translational reprogramming. Emerging evidence suggests that condensates, particularly PBs, intersect with many of these layers, functioning as versatile hubs that integrate and store information about prior stress exposures (87,88). Strikingly, recent findings establish that biomolecular condensates direct cell fate decisions across vertebrate species through selective RNA sequestration, likely revealing that condensate-mediated memory extends beyond stress responses to fundamental developmental transitions (89). Below, we present a concise overview of these principles, emphasizing the convergence of distinct molecular processes into a multitiered cellular memory system. Hence, using PBs as a model system, core principles of condensate-mediated memory can be delineated; these are broadly generalizable and may extend to other condensates and cellular contexts, as mentioned above, across diverse organisms.

## Information feed: The write phase of a condensate

Priming reprograms the transcriptome to establish cellular memory by modulating two distinct classes of memory-associated genes: type I genes maintain elevated transcript levels after an initial stress event, sustaining high expression even after recovery, thereby effectively “remembering” the original encounter. Type II genes do not maintain elevated basal levels but exhibit faster and stronger reinduction upon subsequent stress exposures, enabling a swifter adaptive response. For instance, heat shock factor A2 (HSFA2) orchestrates the regulation of both gene classes by modulating promoter occupancy and nucleosome positioning, thereby remodeling the transcriptional landscape (90). These stress-responsive transcripts are prime candidates for recruitment into PBs, likely as client molecules, where they can be selectively sequestered and protected from degradation or translation. Such compartmentalization constitutes a layer of translational memory, allowing rapid synthesis of key stress-response proteins upon reexposure. Therefore, PB-mediated storage effectively links chromatin-based transcriptional memory with post-transcriptional RNA handling in the cytoplasm.

For transcripts to be incorporated into PBs as clients or scaffolds, they must possess selective molecular features or signals that direct their recruitment or entrapment within condensates. One plausible mechanism involves the conditional association of PBs with the nuclear pore complex (NPC) (91,92). Nuclear basket proteins such as translocated promoter region (Tpr) and nucleoporins have roles beyond nucleocytoplasmic transport including chromatin organization and gene expression regulation. These filaments may provide structural platforms facilitating conditional anchoring or transient association of cytoplasmic or nuclear condensates such as PBs. This spatial coupling would enable efficient capture of newly exported transcripts at the nuclear exit, linking nuclear export directly to cytoplasmic RNA regulation (93,94). This spatial coupling would facilitate rapid and targeted sequestration of transcripts into PBs, linking nuclear export to cytoplasmic RNA regulation. As PBs fill, their volume may increase sufficiently to promote detachment from NPCs, potentially mediated by avid interactions with the cytoskeleton or endomembrane systems (27,91,95,96,97,98).

Interestingly, the NPC itself exhibits gel-like condensate properties, suggesting that the PB-NPC interaction should be conceptualized as a condensate-condensate interaction (10,25,90,99,100,101). This mirrors observations of SG-PB associations in various systems, including plants, supported by compositional analyses identifying NPC components within PB proteomes (102). While direct experimental evidence for NPC-mediated recruitment remains limited, several studies demonstrate that transcript features, including RBP recognition motifs, RNA modifications, and localization signals, contribute to selective PB association (37,103).

Another plausible mechanism for PB incorporation involves epitranscriptomic modifications, analogous to epigenetic DNA marks but at the RNA level (Fig. 1 *B*). In particular, *N*^6^-methyladenosine (m^6^A) could serves as a dynamic molecular tag for transcript-client selection. The m^6^A system operates as a coordinated read-write-erase module: writers, including the methyltransferase A/methyltransferase B (MTA/MTB) complex and accessory subunits, the FKBP12-interacting protein 37 kDa (FIP37) and VIRILIZER (VIR), deposit the modification; readers interpret the mark, and erasers remove it when no longer needed (104,105,106,107). Among the readers, YT521-B homology (YTH)-domain proteins, specifically evolutionarily conserved c-terminal region (ECT) 2, ECT3, and ECT4, bind m^6^A-modified mRNAs in the cytoplasm, forming complexes that stabilize these transcripts by recruiting poly(A)-binding proteins (PAB2/PAB4) (108,109,110,111). Conversely, ECT8 under salt stress conditions, promotes decay of m^6^A-modified transcripts through interaction with DCP5 within PBs (112). This modular mechanism offers a dynamic way to selectively regulate transcript storage or degradation. Methylation marks are written on transcripts, interpreted by reader proteins to determine their fate, either stabilization through the ECT2/3/4-PAB axis or stress-induced decay via ECT8, and erased when no longer required. m^6^A modification acts as a molecular signature that promotes condensate formation by enhancing LLPS through multivalent reader recruitment and contributes to percolation-driven network connectivity within condensates (113). This molecular logic integrates epitranscriptomic regulation with thermodynamic phase separation principles crucial for stress-responsive cellular memory. However, conclusive identification of specific RNA features that define PB clients is pending (10), although some features in nonplants have been suggested (114).

Proteins enter PBs through tightly coordinated processes linking transcriptional induction to translation and subsequent recruitment. Environmental stress activates specific genes; their transcripts often receive modifications (e.g., m^6^A) that signal selective handling (115). These transcripts are exported and translated, producing proteins frequently enriched in IDRs that facilitate multivalent interactions. Newly synthesized proteins, plus RBPs associated with their own mRNAs, can be recruited into PBs via interaction motifs or PTMs. Therefore, transcription-induced translation effectively supplies both RNA and protein clients, shaping condensate composition, enabling dynamic stress molecule storage, regulation, and rapid cellular adaptation.

Our group has shown that even brief heat stress triggers extensive remodeling of RNA and protein clients within PBs, far exceeding transcriptome changes alone (10). During remodeling, many stress-responsive RNAs are sequestered into PBs, while others are degraded or translated rapidly (Fig. 1 *D*). This fine-tunes the balance between RNA storage and decay and influences crosstalk with nearby condensates such as SGs, coordinating global cellular stress responses (Fig. 1 *E*). The remodeling mechanism remains incompletely understood; however, transient temperature increases may raise local concentrations of stress RNAs via translational pauses and lower Csat of scaffold/client proteins This could arise via biophysical changes such as increased IDR radii expanding search volume or pH-induced desolvation in stress (which decreases water retention) (116). If stress endures, further accumulation can surpass the percolation concentration Cperc, strengthening internal interaction networks within condensates, stabilizing PBs against dissolution, and forming persistent “memorizing” condensates.

This dynamic formation of PBs can be conceptualized as a Markov jump process, with PBs occupying discrete compositional states and transitioning between them probabilistically based on environmental inputs (86). The environment acts as an input variable, modulating transition rates between states, influenced by factors such as PTMs or temperature. The steady-state probability of occupancy of each state can be viewed as an output classification, decoding stimuli into functional decisions such as RNA storage (memory) or degradation (resetting). These nonequilibrium networks are subject to thermodynamic constraints limiting their ability to distinguish complex inputs or generate nonlinear responses. The increased stress duration is expected to provide additional input multiplicity (M > 1) that can efficiently inform PBs (e.g., pH changes, modifications). For example, if each input modulates only a single transition (input multiplicity M = 1), the system’s response is monotonic, limiting nuanced regulation, only degradation, or storage. Allowing multiedge modulation (M > 1) increases decision-making complexity and responsiveness. Architectures such as serial cascades arising from complex or prolonged stresses can produce switch-like transitions and richer functional outcomes in PBs and condensates.

Importantly, LLPS systems are inherently out of equilibrium, and they benefit from input multiplicity to enhance responsiveness and functional expressivity (86). The increased stress duration is expected to provide additional input multiplicity (M > 1) that can efficiently inform PBs. This perspective reinforces the notion that evolved phase-separating systems are not merely passive thermodynamic assemblies, but optimized information processors, shaped by the constraints and opportunities of nonequilibrium biophysics. In contrast, if phase behavior were driven by a single input (e.g., only temperature) or domain (e.g., a single protein), responses would remain monotonic and largely nonselective; network structure and feedback mechanisms are therefore critical for generating the nonmonotonic, context-dependent behaviors observed in PBs.

In practical terms, PBs may function a bit like a rheostat with multiple settings: depending on whether the room (the cell) has experienced cold, heat, or fluctuating temperatures (stress history), the rheostat “jumps” between different modes, each tuned to regulate the environment differently. Similarly, PBs may shift between RNA-decay-dominated (erase, see below) and RNA-storage-dominated states (writing), thereby tailoring their functional output with greater precision to the specific stress context. This dynamic reorganization is fundamental for enabling nuanced responses to complex, multistress conditions, a phenomenon that requires further investigation (117). This perspective would match the promiscuity of many biological systems, where networks with higher input multiplicity (M > 1; see above) were demonstrably better at encoding and distinguishing three or more input peaks, achieving nearly correct informational capacity (86).

From the above, it is clear that the writing process cannot be simple or uniform. A central mechanism of the writing process is the selective sequestration of mRNAs that can also follow the Markov jump model showing complexity. Under stress conditions, cells exhibit an initial translational pause marked by ribosome stalling at translation initiation codons, resulting in a sharp reduction in protein synthesis (114,118,119,120,121,122,123,124). During this phase, PBs sequester untranslated mRNAs, bridging the gap until translation can safely resume, thus acting as reservoirs that maintain mRNA stability and modulate translation reactivation. This dynamic can also be modeled by Markov jump processes, capturing the probabilistic nature of translation reinitiation after stress-induced pauses. PBs, as well as other condensates, can store various RNAs that are not immediately needed for protein synthesis during stress. These stored RNAs are often stress specific, allowing them to be reused upon future encounters with the same or similar stress, thereby contributing to memory. This is part of a dynamic mRNA cycling process, where mRNAs, at certain stages of the stress, can move between polysomes (i.e., a proxy of active translation), SGs, PBs, or even other condensates (e.g., the NPC suggested above). Within PBs, mRNAs are held in a translationally inactive state, which serves a dual purpose: conserving cellular resources and prioritizing the synthesis of proteins essential for stress adaptation (10).

In addition to protein-coding transcripts, noncoding RNAs, including miRNAs and lncRNAs, contribute to cellular memory (37,103,125,126,127). Components of the RNA silencing machinery, such as dicing bodies and Argonaut-bound complexes, dynamically traffic to PBs and continue to mediate post-transcriptional gene silencing. Hence, these processes integrate RNA-based regulation with condensate-mediated memory mechanisms (see also (98)). These interactions create a multilayered regulatory network in which PBs not only store and release specific mRNAs but also coordinate broader RNA-based control, enabling fine-tuned, stress-history-dependent responses.

The writing process in PBs also encompasses a proteomic and metabolomic dimension, adding an additional complexity layer to stress memory. Certain PB-resident proteins undergo phase separation and can maintain a defined structural or compositional state after a stress trigger, effectively acting as “biochemical bookmarks” that encode past stress or metabolic conditions and bias future PB behavior. For example, heat-stress-associated 32-kDa protein (HSA32) is retained after stress and contributes to the maintenance of heat memory in plants (24,128). Although direct evidence for the role of secondary metabolites in PB assembly is lacking in plants, metabolomic profiling of SGs indicates the selective accumulation of nucleotides, amino acids, and lipids (129,130). Stress-induced specialized metabolites could further influence PB dynamics by altering cellular viscosity, pH, and redox balance (131,132), which in turn can shift critical concentrations (Csat, Cperc) and material properties, affecting condensate stability and memory retention. Viewed through a thermodynamic and Markov jump framework, these proteomic and metabolomic states can be conceptualized as discrete nodes or “states.”

While PBs are well characterized in yeast and mammalian systems, several aspects of their role in stress memory remain underexplored. Direct evidence for selective storage of specific stress-responsive mRNAs, such as heat shock transcription factors, within PBs is still limited. Similarly, the contribution of PB-resident proteins as biochemical bookmarks and their regulation by certain modifications (e.g., phosphorylation, methylation) has not been systematically addressed. Additionally, the interplay between PBs and other condensates, including SGs, and how these interactions influence RNA routing and memory formation, remains to be clarified. Finally, quantitative measurements linking PB composition, material properties, and cellular memory outcomes under varying stress regimes are scarce. Addressing these gaps will be essential to fully understand how PBs and other condensates integrate multilayered molecular signals to write stress history.

## The read phase of the condensate

Once information is written in PBs as client proteins, RNAs, and possibly metabolites, it must remain readily accessible. Under moderate stress, client molecules can be selectively released from PBs, rapidly supplying functional proteins and RNAs essential for adaptation. In contrast, during severe or prolonged stresses distinct from prior experiences, PB scaffolds themselves may dismantle, causing condensate dissolution and the release of long-term stored molecules. Concurrently, new PBs form de novo in a new “write” phase or through the help of other condensates such as SGs (Fig. 1, *D* and *E*). Conversely, when stress subsides and conditions normalize, specifically when concentrations drop below Csat, partial condensate dissolution occurs, releasing certain transcripts while retaining others within a dense, percolated core. This selective retention and release encode a graded, thermodynamically governed cellular memory of the stress event.

Molecular memory is best understood as a dynamic, adaptive process rather than a fixed state. Consider a rapid, transient stress cue lasting seconds to minutes: PBs rapidly load transcripts present in the cytoplasm along with their bound RBPs. Simultaneously, some preexisting PBs dissolve, releasing previously stored transcripts and proteins for immediate use (10); this transcriptome could be affected also by the time of the day that the release takes place (133). This nonspecific “first wave” response equips the cell with a broad arsenal to counter sudden perturbations efficiently, surpassing reliance on de novo transcription. Viewed through the thermodynamic and nonequilibrium lens, these compositional shifts correspond to transitions between discrete states (nodes) in the Markov jump network.

If the stress persists, cellular responses transition from this rapid, nonspecific phase to a targeted, adaptive phase. Newly formed PBs accumulate transcripts selectively induced by ongoing stress through continuous transcription and selective incorporation. Our group has shown that both proteins and their cognate RNAs progressively accumulate in PBs with increasing stress duration (10). This model proposes two functional PB types: 1) preformed PBs, which act as “buffers” for immediate, nonspecific responses by releasing generic stress-responsive transcripts and proteins and 2) de novo PBs, which function as “adaptive memory units,” selectively retaining transcripts and proteins tailored to the specific stress if it persists. In the Markov jump framework, preformed PBs occupy rapidly accessible states, whereas de novo PBs represent stabilized memory states arising under prolonged stress exposure. An analogy is that preformed PBs function like a home first-aid kit stocked for miscellaneous injuries, while de novo PBs resemble tailored prescription medicines prepared after diagnosis. Thus, PBs and likely other condensates serve as crucial first-line defenders, bridging immediate responses with longer-term, finely tuned adaptations.

PB dynamics are orchestrated by the cytoskeleton, which regulates their assembly, movement, and spatial organization (11,91). Actin filaments and microtubules serve complementary roles: microtubules can scaffold PB docking with SGs, facilitating the reversible exchange of mRNAs and their stabilization, particularly during early stress responses (134). Meanwhile, actin-driven motility redistributes PBs to subcellular locales where selective preservation of transcripts and decay factors occurs, effectively creating “hotspots” for rapid release of their contents and translational reactivation (135). Accordingly, proximity interactome profiling has revealed interactions between PB components and actin-nucleating complexes, such as the suppressor of cAMP receptor-WASP-family verprolin-homologous (SCAR-WAVE) and actin-related protein 2/3 (ARP2/3) complexes, suggesting that actin-mediated transport helps determine PB positioning and connectivity (91) (Fig. 1 *E*).

We cannot discount the possibility that microtubules could exert similar functions, as was shown for reliquification of microtubule-associated protein 65 (MAP65) condensates in vitro (136). This process increases the mobility of MAP65 molecules within the condensate and returns them to solution. This observation implies a feedback loop: condensate formation influences microtubule organization and, in turn, microtubule growth alters the properties of condensate material. It is worth noting that PBs accumulate many microtubules and actin-nucleating factors (10), and a similar feedback loop could be envisioned.

Furthermore, these cytoskeletal interactions appear to interface directly with the percolated network of PBs, with complexes such as SCAR-WAVE acting as “clients” that can locally remodel or partially disassemble PBs (10), thereby releasing proteins and RNAs for immediate cellular responses. This dynamic highlights how client-scaffold coupling and cytoskeleton-mediated forces can transiently tune PB dynamics. However, a major open question remains: it is still unclear how “aged” or more solid-like PBs with “long-term memories”, which have potentially undergone partial hardening or gelation, can efficiently release their stored content. One scenario, is that first the younger PBs will be dismantled being in a liquid-like and hence “softer” state (with more recent memories), while, as suggested above, later old PBs will follow (likely with more universal core stress responses). Understanding the mechanisms behind content mobilization from long-lived condensates with perhaps older foundational memories is critical for linking PB dynamics to long-term memory.

## The erase phase (resetting memory)

For memory to remain adaptive, it must be reversible, necessitating the active clearance or resetting of stored information to prevent maladaptive persistence (Fig. 1 *D*). A crucial aspect of reversibility is the regulated erasure of PB composition when stress abates and the targeted degradation of molecular clients. Cellular proteostatic surveillance mechanisms provide this deletion layer, selectively removing aged, damaged, or dysfunctional PBs. As PBs age, their material state often shifts from dynamic, liquid-like assemblies to more solid or gel-like conditions, entrapping PBs in metastable structures that promote dissolution and clearance. These physical changes enhance recognition by cellular quality control machinery, including molecular chaperones (99,137). For example, proteostatic pathways such as aggresome formation and aggrephagy effectively identify and target rigid or aggregated PBs for degradation (99). Solidified condensates can sequester aggregation-prone proteins, thereby protecting cellular functions during stress. These states foster interactions with chaperones such as heat shock protein (Hsp) 70 and Hsp90, and disaggregases, facilitating refolding or removal and promoting clearance. Such metastable, solvent-exposed states render solid PBs accessible to surveillance pathways marking them for elimination (138).

Simultaneously, under stress, PBs recruit decay-promoting factors such as DCP1/2 and XRN4 (139,56). These condensates participate actively in selective mRNA decay, including nonsense-mediated decay and other degradation pathways, shifting PBs from storage-dominant to clearance-centric states (140,141,142). This involves partial PB disassembly and reassembly into more liquid-like forms capable of recruiting decay machinery and “resetting” memory modules, thus replacing outdated assemblies with newly adapted ones reflecting current environmental conditions (10,91). Such compositional changes can be triggered by new client RNAs and proteins, often multivalent and IDR rich, induced by stress, which destabilize preformed percolated networks enforcing comprehensive remodeling (143).

The transition from the storage (S) to decay (D) state is not instantaneous but depends on the cumulative cellular experience of stress, which integrates both intensity and duration. We propose that the transition rate $k_{S\to D}\left(t\right)$ follows a saturating exponential form: $k_{S\to D}\left(t)=\alpha (1-e^{-\gamma \int_{0}^{t}I\left(\tau \right)d\tau }\right)$. This functional form is derived from the underlying biochemistry of stress sensing and response: the integral $\int_{0}^{t}I\left(\tau \right)d\tau$ represents the cumulative “stress dose,” a concept well established in toxicology and stress physiology (144). It captures the intuitive notion that a prolonged, mild stress, and a brief, intense stress, can have similar effects if their integrated intensity is equal. The exponential saturation models the cooperative kinetics of the cellular response. The parameter *γ* scales the system’s sensitivity and is influenced by the effectiveness of stress signal transduction (e.g., kinase activation cascades such as the MAPK pathway) (145). Initially, the system resists switching states (low rate) but, as cumulative stress builds, the transition rate increases rapidly before saturating at a maximum hypothetical value *α*. The maximum rate *α* represents the limiting maximum rate of transition, constrained by the biophysical properties of the condensate (e.g., molecular mobility within the gel-like core) and the catalytic turnover rates of the major decay enzymes (e.g., DCP2). The reverse transition, from decay back to storage, is driven by recovery processes (e.g., phosphatase activity, synthesis of new components or even contrasting kinases (146,147)) when stress abates. For simplicity, we model this recovery rate $k_{D\to S}$ as a constant *β*. The time evolution of the probability $P_{S}\left(t\right)$ of being in the storage state is then governed by the master equation: $\frac{dP_{S}}{dt}=-k_{S\to D}\left(t\right)P_{S}\left(t\right)+\beta \left(1-P_{S}\left(t\right)\right)\text{.}$ The parameters *α*, *β*, and *γ* are not abstract but are governed by the physical principles of LLPS. For instance, *γ* is sensitive to the concentration, for example, of stress-activated kinases regulating PBs (e.g., MAPKs (148)) relative to their critical concentration for clustering ($C_{\text{sat}}^{\text{kinase}}$). Similarly, *α* is limited by the molecular mobility within the condensate, which is dictated by its material state (liquid versus gel). This model therefore integrates the stochastic kinetics of cellular decision-making with the fundamental thermodynamics of biomolecular condensates. This framework makes the following testable predictions: the switching kinetics should show a delay followed by a saturating response to a step stress and the model predicts “priming”: a brief, subthreshold stress that increases ∫I(τ)dτ should lead to a faster transition upon a second stress. Mutations that affect kinase activity (or in general PBs regulators altering *γ*) or condensate material properties (altering *α*) should produce quantitatively predictable changes in PB dynamics and translational outcomes (Fig. 1 *E*). Validating this model requires future work measuring these transition rates in live cells under controlled stress conditions.

In a Markovian framework, PTM-based regulations act as dynamic “rate modifiers” that increase or decrease the likelihood of a jump into decay versus storage modes. We anticipate that these kinetic regulations will exhibit nonlinear behavior due to several intrinsic biological factors. These regulations exhibit inherent nonlinearities arising from cooperative kinetics (modeled by Hill functions), enzyme saturation (Michaelis-Menten dynamics), and feedback loops within signaling network dynamics (149,150). Additionally, positive and negative feedback loops within signaling and regulatory networks amplify or dampen responses in a nonlinear manner. Molecular crowding and condensation further modulate reaction environments and intermolecular interactions (151,152), reinforcing nonlinear behavior. This complex interplay allows PBs to integrate diverse environmental cues probabilistically, facilitating dynamic recalibration of transcript preservation and degradation as stress conditions evolve.

A comprehensive experimental validation of this framework requires a multipronged approach designed to directly measure its parameters and test its predictions. A primary objective is to track memory state transitions in living cells. This could be achieved through multicolor live-cell imaging of *Arabidopsis* lines expressing fluorescently tagged PB components (e.g., DCP1-GFP, XRN4-mCherry) during defined stress-recovery cycles. By quantifying the kinetics of transitions between storage and decay states in individual condensates over extended periods (24–72 h), one could directly measure the rate constants (*α*, *β*) and validate the proposed dependence of the storage-to-decay transition on cumulative stress dose.

A second critical goal is to empirically determine the hierarchical relationship between critical concentration thresholds. Combining quantitative proteomics of isolated PBs with systematic stress titrations would allow measurement of client and scaffold concentrations within condensates as a function of stress intensity. This approach would test the model’s prediction that phase separation (Csat) precedes percolation (Cperc), which in turn establishes a functional memory state. The model’s core postulate, that PBs store specific, retrievable information, can be tested by challenging primed plants with either the same stressor or a novel one. Comparing the transcriptional response and PB dynamics in each scenario would reveal whether memory is stress-type specific and is associated with a unique molecular signature within the condensate. Furthermore, the concept of input multiplicity (M) can be probed by applying combinatorial stresses and using single-cell analysis to determine if PB compositional complexity scales with the number of distinct input signals, thereby enhancing information encoding capacity.

The proposed link between material state and memory function can be directly investigated by correlating physical properties with storage duration. Techniques such as fluorescence recovery after photobleaching could measure molecular mobility within PBs during different memory phases, testing the prediction that gel-like states (low mobility) correlate with extended memory retention. Finally, causal validation of the model requires targeted perturbations. Genetic or pharmacological manipulation of key nodes, such as kinase activity or client molecule concentrations, should produce quantitatively predictable changes in transition rates and memory behavior, for instance, delaying the storage-to-decay transition upon modulating, for example, PTMs. This integrated experimental framework transforms the theoretical model into a set of testable hypotheses, paving the way for a quantitative, predictive understanding of condensate-mediated memory.

## Future perspectives

**Figure 2:**
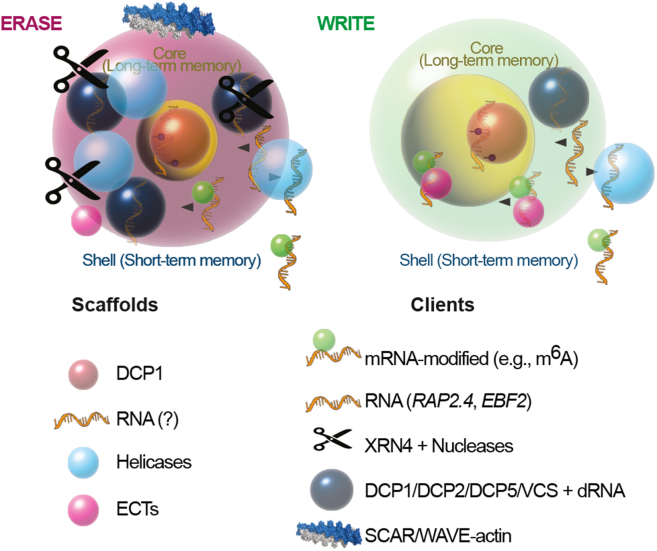
Conceptual model of two PBs states and associated molecular components. An ERASE mode (*left*) enriched in nucleases and decay machinery at the liquid periphery, and a WRITE mode (*right*) associated with RNA protection, modification, and regulatory storage. Each condensate displays a core region representing long-term molecular memory (large for the WRITE phase) and a shell corresponding to short-term memory (large for the ERASE phase). Key scaffold components (DCP1, RNA, helicases, and ECT proteins) and client molecules (including modified mRNAs such as m^6^A, specific transcripts such as *RAP2.4* and *EBF2*, XRN4, and other nucleases, decapping factors DCP1/2/5/VCS with RNA, and SCAR/WAVE-actin networks) are indicated.

The integration of thermodynamic principles, mathematical modeling, and condensate biology presented in this review reveals PBs as sophisticated cellular systems that may function as molecular memory repositories. We summarize some of the relevant PB components relevant to this framework in Fig. 2. Our framework demonstrates how the fundamental physics of phase separation, governed by critical concentrations and material state transitions, could enable PBs to encode stress-responsive information through selective molecular sequestration, maintain this information through gel-like network stabilization, and retrieve it through controlled liquefaction and client release. The mathematical model provides quantitative predictions linking condensate physical properties to memory performance, while the proposed experimental approaches offer concrete pathways for validation. This synthesis suggests that condensate-mediated memory represents a previously underappreciated layer of cellular information processing that operates alongside genetic and epigenetic mechanisms to enhance plant stress resilience.

Advanced methods such as high-resolution imaging and single-condensate proteomics will be instrumental to decipher the biophysical rules governing client selection and residence time. Key questions include understanding how condensates such as PBs selectively gate mRNA fate, deciding whether to store, degrade, or release transcripts for translation poststress, and whether biochemically distinct condensate subtypes exist for short- versus long-term memory roles. Furthermore, a critical future direction is the quantitative validation of theoretical models, such as the Markov jump process framework proposed here. This requires advanced live-cell imaging to track PB composition and dynamics in real time, coupled with computational methods to infer transition rates. Techniques such as fluorescence recovery after photobleaching can provide experimental estimates of parameters such as molecular mobility (*α*), while mutational analyses of key kinases and phosphatases can test predictions about *γ* and *β*. Ultimately, building a quantitatively predictive model of condensate-mediated memory will require this close integration of theory, experimentation, and computational biology.

While condensates have traditionally been viewed from a “protein-centric” perspective focusing on scaffold proteins, emerging evidence highlights the critical role of RNA structural dynamics in cellular memory. Environmental stress can induce conformational switches in RNA molecules; PBs and their resident RBPs may stabilize these alternative structures and act as catalytic hubs, templating them on nascent RNAs in a prion-like fashion (153). This suggests that PBs function not only as transient sequestration sites but as propagators of heritable structural memory, influencing cellular adaptation and future stress responses.

Another thrilling frontier lies in elucidating how dynamic cytoplasmic condensates communicate with nuclear memory systems. This concept extends to epigenetic memory, as PBs may indirectly modulate chromatin states by degrading noncoding RNAs required for chromatin modifications, thus erasing epigenetic marks and resetting gene expression programs. Parallels with *Caenorhabditis elegans* germ granules, which organize RNA-based inheritance and segregate small RNAs, suggest that plant PBs or related condensates might similarly package stress-responsive small RNAs into egg cells, providing a mechanistic basis for maternal inheritance of stress memory (154,155). Similarly, plant PBs or related condensates could also package stress-responsive RNAs into the egg cell, providing a compelling explanation for maternal inheritance (154,155).

Finally, PBs emerge as master regulators of transcript abundance, providing buffering against stochastic noise inherent in gene expression. In fungi and metazoans, cytoplasmic exonuclease Xrn1 couples mRNA decay to transcription via nuclear feedback (156,157). Although this precise nuclear feedback remains unproven in plants, the *Arabidopsis* XRN4 exonuclease fulfills a central PB-associated decay function. Here, transcript buffering is achieved by dynamically balancing mRNA decay and translation efficiency (158). Beyond decay, PBs serve as dynamic reservoirs, strategically storing and releasing RNAs to smooth transcriptional fluctuations (159). Unraveling these intricate systems will advance our understanding of how organisms such as plants finely tune cell and nuclear memories with remarkable precision, a cornerstone of their resilience.

## Acknowledgments

This work received funding from 10.13039/501100000781European Research Council Consolidator Grant PLANTEX, 101126019, 10.3030/101126019 (to P.N.M.). Views and opinions expressed are those of the authors only and do not necessarily reflect those of the European Union or the European Research Council Executive Agency. Neither the European Union nor the granting authority can be held responsible for them.

## Author contributions

S.M. performed the initial literature survey and drafted the initial manuscript. P.N.M. acquired funding, conceptualized the framework, the thermodynamic and kinetic models, developed the Markov jump mathematical framework, formulated the condensate memory phases (write-read-erase), designed the analytical approach linking phase separation to information storage, created the figures, and critically revised the manuscript for intellectual content. Both authors contributed to the final editing and approved the submitted version.

## Declaration of interests

The authors declare no conflict of interests.

## Declaration of generative AI and AI-assisted technologies in the writing process

During the preparation of this work the authors used Claude and ChatGPT to edit for readability. After using these tools, the authors reviewed and edited the content as needed and take full responsibility for the content of the publication.
